# Atomic scale modelling of hexagonal structured metallic fission product alloys

**DOI:** 10.1098/rsos.140292

**Published:** 2015-04-01

**Authors:** S. C. Middleburgh, D. M. King, G. R. Lumpkin

**Affiliations:** 1 Institute of Materials Engineering, Australian Nuclear Science and Technology Organisation, Locked Bag 2001, Kirrawee DC, New South Wales 2232, Australia; 2 Department of Nanomaterials, University of Technology, Sydney, New South Wales, Australia

**Keywords:** high-entropy alloy, fission product, nuclear fuel performance and safety, density functional theory

## Abstract

Noble metal particles in the Mo-Pd-Rh-Ru-Tc system have been simulated on the atomic scale using density functional theory techniques for the first time. The composition and behaviour of the epsilon phases are consistent with high-entropy alloys (or multi-principal component alloys)—making the epsilon phase the only hexagonally close packed high-entropy alloy currently described. Configurational entropy effects were considered to predict the stability of the alloys with increasing temperatures. The variation of Mo content was modelled to understand the change in alloy structure and behaviour with fuel burnup (Mo molar content decreases in these alloys as burnup increases). The predicted structures compare extremely well with experimentally ascertained values. Vacancy formation energies and the behaviour of extrinsic defects (including iodine and xenon) in the epsilon phase were also investigated to further understand the impact that the metallic precipitates have on fuel performance.

## 2. Introduction

Investigation of fission product behaviour will improve the safe and efficient use of nuclear fuel in both current and future nuclear reactors. The soluble, volatile, noble gas and oxide forming fission products have been the focus of a great deal of recent work, both theoretical [1–12] and experimental [13–16]. The metallic precipitates have had less focus, potentially as a consequence of the scarcity of the elements that make up the precipitates and the deviation in modelling methods (mainly due to their metallic nature). Despite these difficulties, limited work has been carried out [17–19]. The metallic fission product precipitates can contain a number of elements (including Mo, Tc, Ru, Pd, Ag, Cd, In, Sn and Sb) which make up approximately 27% of the fission yield in a 3.2% enriched pressurized water reactor fuel at 2.9% burnup [7]. Not only have metallic precipitates been observed in oxide fuels, but they also form in carbide fuels (although the composition is known to vary significantly [20]) and would be expected to form in nitride and silicide fuels as well.

A major metallic phase that forms in oxide fuel is the epsilon phase, known also as the white phase, five-metal particles or noble metal particles [21–27]. The phase has a hexagonal-closed packed (HCP) crystal structure and forms over a wide range of stoichiometries in the Mo-Pd-Rh-Ru-Tc system [23]. Due to the similarities of Ru with Tc and Pd with Rh in metallic radius and chemistry, this system has been simplified by Kleykamp *et al.*, who considered the Mo-(Pd,Rh)-(Ru,Tc) pseudo-ternary system [22,23].

Molybdenum is the most readily oxidized of the epsilon phase constituent elements and as such will preferentially react with any excess oxygen available in the system [22,23]. Oxygen is released in oxide fuels as a consequence of fission processes due to the lower overall oxidation state of the daughter products (e.g. Kr^0^ and Ba^2+^ produced by a U-235 fission) [28]. Oxygen will readily react with Mo in the epsilon phase forming, for example, MoO_2_ (further reacting with SrO and BaO to form (Sr,Ba)MoO_3_ grey phases) [29]. These processes will reduce the concentration of Mo in the metallic precipitate. As such, the Mo content is a good measure of burnup, and the Ru : Mo ratio is used as a standard measure [23].

The ratio of (Ru,Tc) to (Rh,Pd) that is produced due to fission processes is approximately 7 : 3 [7,19,22]. Maintaining this ratio of (Ru,Tc) to (Rh,Pd) with increasing concentrations of Mo is reported to produce a number of different phases. Low concentrations of Mo less than 5% at 1700^°^*C* will produce a mixed *α* (FCC) and *ϵ* (HCP) phase [22]. Greater amounts of Mo content will produce a single-phase HCP structure with increasing lattice parameter ‘a’ values with increasing Mo content. This HCP phase exists from low concentrations of Mo to approximately 50 at.% Mo. Beyond this, increased amounts of Mo promote what is termed *σ* phase formation (a notation that normally relates to the CrFe structure but in this case is more likely to be the Cr_2_Fe Laves structure) and then a *β* (BCC) phase is formed which becomes single phase with greater than approximately 80 *at*.% Mo [22]. Many phase diagrams assume identical behaviour of Tc with Ru (substituting Ru for Tc). The ratio between the Ru and Tc varies as a result of the radioactivity of Tc, but the ratio in fresh fuel is often reported to be approximately 2Ru : 1Tc [22,23,26].

This work focuses on the effect of varying Mo concentrations in the HCP epsilon phase. The structure and stability of the phase with respect to the constituent metallic elements is initially investigated. Systems that omit Tc are considered and compared to experimental phase diagram data before investigating the impact of Tc additions, simulating a more realistic composition. The defect behaviour in the phases with varying Mo content is then examined to understand the accommodation of intrinsic and extrinsic defects (including the fission products iodine, tellurium, xenon and tin).

## 3. Methodology

Atomic scale calculations were carried out using density functional theory (DFT) to describe the inter-atomic bonding. Compounds in the Mo-Pd-Rh-Ru-Tc system are metallic and as such plane wave DFT, as implemented by the Vienna Ab-initio Simulation Package [30,31], was chosen as a suitable method [32]. Other methods such as empirical potential descriptions of the bonding [33,34] may also prove useful in future studies but potentials for this system are yet to be developed.

As the bonding is metallic, special attention is given to the smearing method and width: the Methfessel–Paxton method was used with a width of 0.125 eV (giving an accuracy of more than 10^−3^ eV per supercell). A 3×3×2 *γ*-centred *k*-point grid and a cut-off energy of 500 eV were employed, giving an accuracy greater than 10^−3^ eV. Pseudopotentials with the highest number of valence electrons in the supplied VASP library ware chosen for each element (Mo with 12, Tc with 13, Ru with 14, Rh with 15 and Pd with 16) and the GGA-PBE exchange correlation was used throughout the study [35]. Each supercell was fully geometry optimized under constant pressure. Careful consideration was given to the volume and shape of the unit cell during the relaxation as metastable geometries far from the equilibrium value (less favourable and higher energy) were occasionally produced. The stopping criterion for the self-consistent field steps was 10^−4^ eV, whereas the stopping criterion for the geometry optimization was 10^−3^ eV. The total forces acting upon the system were calculated to be less than ±−0.005 eV Å ^−1^ and ±−0.02 eV Å ^−1^ on each atom; these are acceptable values given the random nature of the alloys being investigated.

The solid solutions were produced by randomly populating an HCP lattice containing 54 lattice sites with various numbers of each element depending on the target stoichiometry. Ten different supercells of each stoichiometry were produced to provide an acceptable statistical sample (*σ*<0.01 eV). The compositions are summarized in table 1. This method was used in past work to investigate FCC high-entropy alloys [32] and compared well with other studies using more traditional methods for random systems including using the special quasi-random structures method [36,37]. For the FCC high-entropy alloy, key properties such as lattice constants [38] were reproduced well within the errors of the method.

**Table 1.** The composition of the systems providing different concentrations of Mo in a 54 atom supercell of epsilon phase.

	number in supercell				
Mo concentration (at.%)	Mo	Pd	Rh	Ru	Tc
*Mo-Pd-Rh-Ru system*					
0	0	8	8	38	—
3.7	2	8	8	36	—
11.1	6	7	7	34	—
14.8	8	7	7	32	—
25.9	14	6	6	28	—
37.0	20	5	5	24	—
51.9	28	4	4	18	—
*Mo-Pd-Rh-Ru-Tc system*					
0	0	8	8	25	13
3.7	2	8	8	24	12
11.1	6	7	7	23	11
14.8	8	7	7	21	11
25.9	14	6	6	19	9
37.0	20	5	5	16	8
51.9	28	4	4	12	6

Defect formation energies were calculated using a well-accepted method employed in a number of similar studies [32,39,40]. Defect formation energies are useful for calculating the drive for intrinsic defect production allowing some understanding into the drive for secondary phase formation and phase stability. The defect formation enthalpy for a vacancy can be calculated by taking the difference between a perfect supercell and a supercell containing a vacancy and adding the energy of the removed species' mono-atomic metal. This can be written as the following for Mo vacancy formation where body centred cubic Mo metal is produced as a result of vacancy formation in the alloy (using a modified Kröger–Vink notation [10,41]):

3.1 $${\text{Mo}}_{A}\to V_{A}+\text{Mo(s)},$$

where Mo_A_ is a Mo on an HCP lattice site in the epsilon phase and a V_A_ is the vacant lattice site produced as a consequence of the reaction. The processed data, including the formation and solution energies presented in this work, are available in the electronic supplementary material.

## 4. Results

### 4.1 Variation of Mo in the epsilon phase

#### 4.1.1 Ru-Pd-Rh-Mo system

In a similar manner to the phase diagrams presented by Kleykamp *et al.* [22,23], a set of epsilon phase compositions were investigated that comprised equimolar amounts of Rh and Pd metals, combined with Ru metal such that the Ru : (Pd,Rh) was approximately 2.33. Additions of Mo metal were then sequentially added such that the Mo concentration varied from 0 at.% to 51.9 at.%. This provides a range of compositions that are expected to be observed from a variety of nuclear fuel environments.

As the supercells contain 54 atoms, the Ru : (Pd,Rh) ratio could not be maintained exactly: seven stoichiometries were produced with their Ru : (Pd,Rh) ratio varying between 2.25 and 2.43 (see table 1). The formation enthalpies of these compositions were determined by comparing the solid solution to the individual metal phases. Reaction (4.1) gives an example where the concentration of Mo is 11.1 at.%:

4.1 $$6\text{Mo}+7\text{Pd}+7\text{Rh}+34\text{Ru}\to {\text{Mo}}_{6}{\text{Rh}}_{7}{\text{Rh}}_{7}{\text{Ru}}_{34}.$$

**Figure 1.**
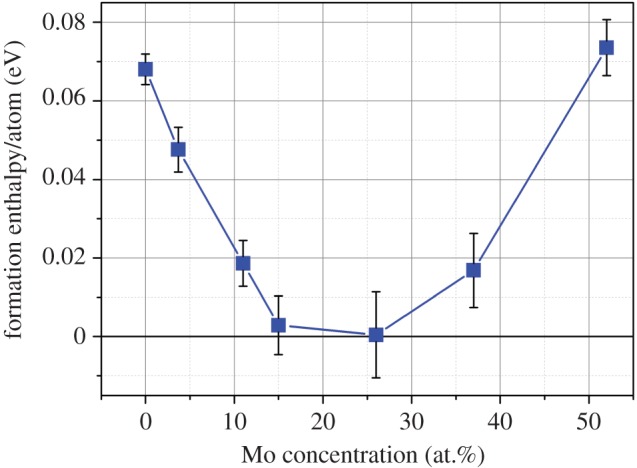
Variation in formation enthalpy of the Ru_7_Rh_1.5_Pd_1.5_Mo_*x*_ HCP phase with respect to the constituent metallic systems.

The enthalpies of formation were normalized per atom for ease of comparison and are presented in figure 1. The average enthalpies calculated are all positive, the most favourable being the 25.9 at % Mo composition. As the system is highly disordered and can be treated as an ideal solution, the configurational entropy term will be an important factor, stabilizing the alloys at higher temperatures. The configurational entropy can be estimated using the Boltzmann equation [42]:

4.2 S=kln⁡Ω,

where *S* is the configurational entropy, *k* is the Bolzmann's constant (8.617×10^−5^ eV *K*^−1^) and *Ω* is the configurational term which is dependent on the number of sites available to the number of elements in the system. This can be calculated by using the general equation

4.3 $$\Omega =\frac{N!}{n!(N-n)!}.$$

In the case of one supercell containing 38 Ru, 8 Rh and 8 Pd, the *Ω* term would be calculated as

4.4 $$\Omega =\frac{54!}{38!8!8!}=2.715\times 10^{17}.$$

**Figure 2.**
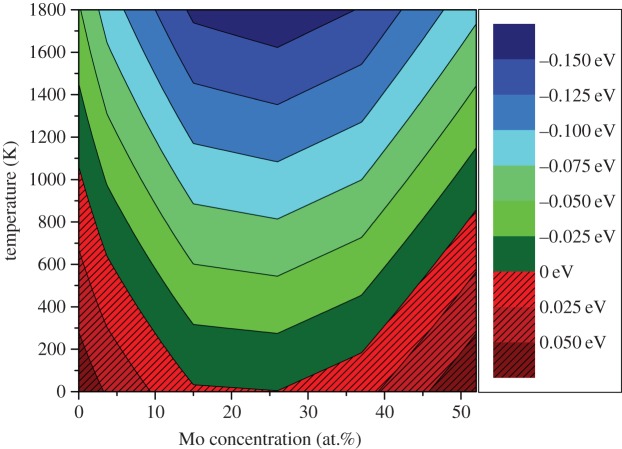
Variation in Gibbs free energy of the Ru_7_Rh_1.5_Pd_1.5_Mo_*x*_ HCP phase with respect to the constituent metallic systems with temperature.

The *Ω* from equation (4.4) provides a configurational entropy of 3.46×10^−3^ eV *K*^−1^ per supercell (or 6.41×10^−5^ eV *K*^−1^ per atom). By including this temperature-dependent entropy term, one can make a good estimate of the Gibbs free energy of the system (although terms such as the vibrational entropy are not considered). Figure 2 reports the change in Gibbs free energy with composition at temperatures between 0 and 1800 K. As the temperature increases, the Gibbs free energy of the system falls. At 1000 K, all formation energies are computed to be negative. It should be made clear that other phases are expected to exist at the extremes of the Mo concentrations considered according to the Mo-Ru-(Rh,Pd) phase diagram by Kleykamp *et al*. [22]. These are not considered in this work so no definitive comment can be made on the stability of the system in relation to the FCC and *σ*-phase that are expected to form.

**Figure 3.**
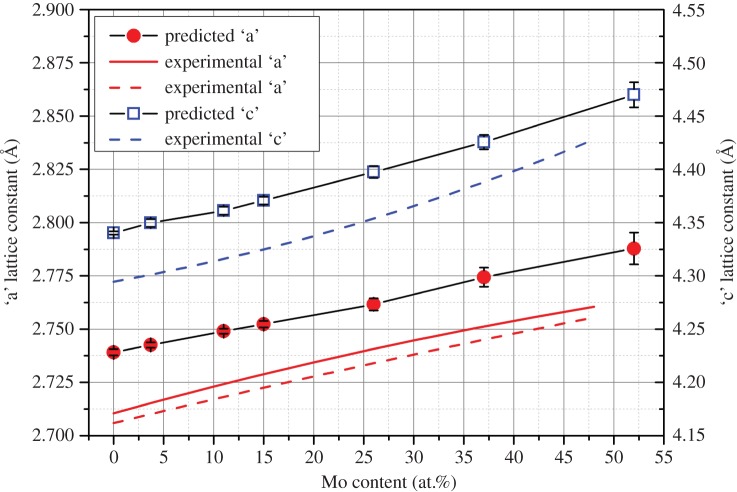
Predicted variation in ‘a’ and ‘c’ lattice constants in the hexagonal close packed structure of Ru_7_Rh_1.5_Pd_1.5_Mo_*x*_. Experimental data of the ‘a’ lattice constant from Kleykamp *et al.* [22] and Kleykamp & Pejsa [24] (solid red line) and the experimental ‘a’ and ‘c’ lattice constants from Ru_7_Rh_3_Mo_*x*_ from Dwight & O'Boyle [43] is also provided (dashed lines).

The lattice parameters of the phases have been predicted to vary in a manner similar to those experimentally measured in past work by Kleykamp *et al*. [22] & Dwight & O'Boyle [43]. Both ‘a’ and ‘c’ lattice parameters were predicted to increase with increasing Mo content. This is to be expected given the larger metallic radius of Mo (1.39 Å) compared to the other elements in the alloy (*Ru*=1.34 Å , *Rh*=1.34 Å and *Pd*=1.37 Å) [44]. Figure 3 reports the changing behaviour. The approximate 1% overestimation of both the ‘a’ and ‘c’ parameters in the system is expected due to the use of the GGA-PBE exchange correlation, which is known to slightly underestimate bonding and overestimate bond distances as a result. The c : a ratio of the hexagonally closed packed system increases from 1.585 to 1.603 with increasing Mo content towards the ideal ratio of 1.633.

#### 4.1.2 Tc-Ru-Pd-Rh-Mo system

Technetium is known to be present in high concentrations in the epsilon phase but was not considered in a number of previous experimental studies as it is radioactive (Tc-99m is a *γ*-emitter and Tc-99 is a *β*^−^-emitter). As Tc has a similar metallic radius and chemistry to both Ru and the other constituents of the epsilon phase, one would not expect any large variation in the stability of the alloy.

**Figure 4.**
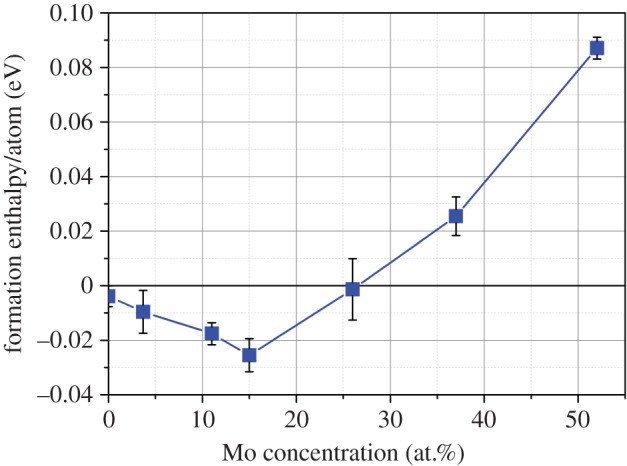
Variation in formation enthalpy of the (Tc_1/3_Ru_2/3_)_7_Rh_1.5_Pd_1.5_Mo_*x*_ HCP phase with respect to the constituent metallic systems.

In a similar manner to the Ru-Pd-Rh-Mo system investigation, we now discuss how the variation of Mo concentration affects the stability in the Tc-Ru-Pd-Rh-Mo system. The ratio of Ru : Tc is maintained at between 1.9 and 2.1, a ratio observed in a number of previous experimental studies [17,22,23,25]. The formation enthalpies for the compositions with increasing Mo content are presented in figure 4.

**Figure 5.**
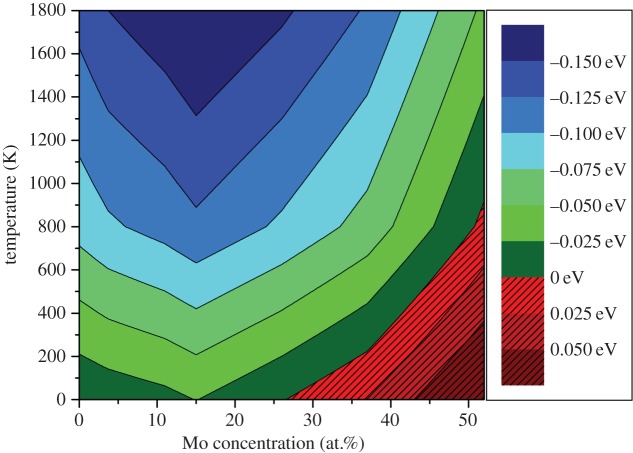
Variation in Gibbs free energy of the (Tc_1/3_Ru_2/3_)_7_Rh_1.5_Pd_1.5_Mo_*x*_ HCP phase with respect to the constituent metallic systems with temperature.

Unlike the system which omits Tc, figure 4 reports a significant proportion of the Tc containing system that has a negative formation enthalpy when formation is considered from the constituent metallic systems. The favourable formation enthalpies were calculated to dominate when the Mo concentration was under approximately 25.9 at.%. The estimate of configurational entropy is included in figure 5, where the stability is shown to increase through a reduction in the Gibbs free energy of 0.075 eV by 900 K.

The lattice parameters of the hexagonal phases containing Tc are very similar to those that omit the element (figure 3). An increase of 0<*x*<0.008 Å in the ‘a’ parameter and 0<*x*<0.017 Å in the ‘c’ parameter is observed for all compositions, which is not unexpected given the slightly larger metallic radius of Tc (1.36 Å) compared with Ru (1.34 Å).

### 4.2 Vacancy formation energy in the epsilon phase

**Figure 6.**
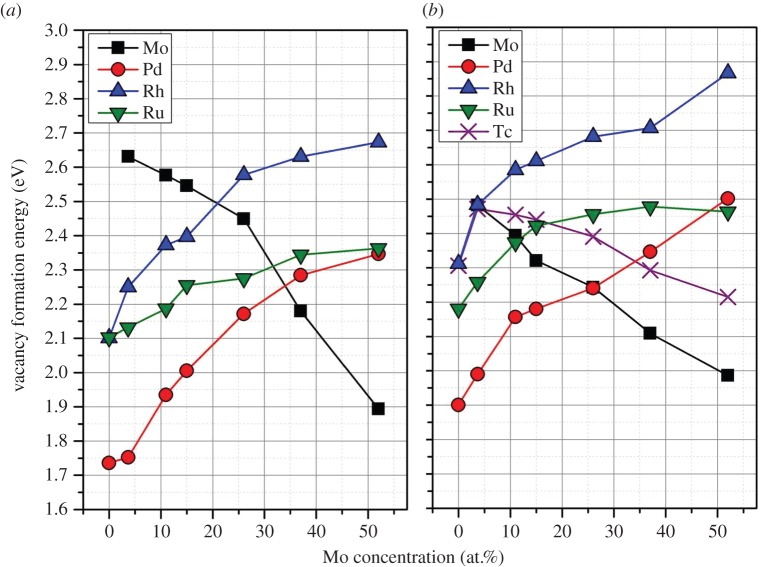
Variation in average vacancy formation energy at each composition as a function of Mo concentration in the epsilon phase which (*a*) omits Tc and (*b*) includes Tc. The standard deviation was less than ±0.04 eV for all data.

In a similar manner to past work investigating the stability of the CoCrFeNi high-entropy alloy [32], we now investigate the drive for vacancy formation in the epsilon phase. Unlike the CrCoFeNi system, all species are predicted to have positive vacancy formation energies, indicating that there is no drive for one element to segregate out of the phase. Figure 6 reports the change in vacancy formation enthalpy for each element as a function of Mo content in the alloy. Both systems indicate that the most preferential element to form a vacancy is Pd at low Mo concentrations and Mo at the higher Mo concentrations.

**Figure 7.**
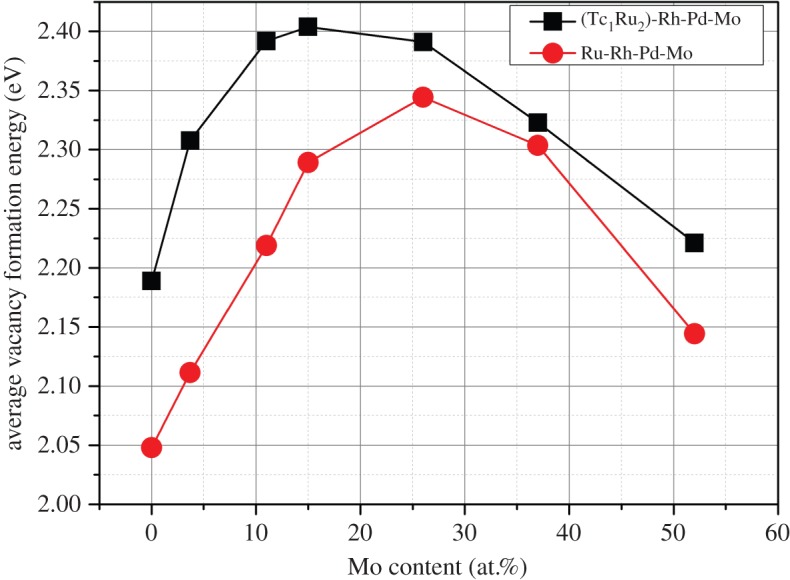
Variation in vacancy formation energy as a function of Mo concentration in Ru_7_Rh_1.5_Pd_1.5_Mo_*x*_ and (Tc_1/3_Ru_2/3_)_7_Rh_1.5_Pd_1.5_Mo_*x*_.

The average vacancy formation enthalpy was calculated from the individual vacancy formation energies. Figure 7 reports a peak of average vacancy formation enthalpy at a Mo content of 25.9 at.% in the system that omits Tc and a peak at a lower Mo concentration of 14.8 at.% in the system that includes Tc. Vacancy formation energies in the Tc-containing system were all higher for a given Mo concentration, and as a result, the equilibrium vacancy concentration will be expected to be lower.

Past work has calculated the vacancy formation energies of V and Cr to be approximately 2 eV, similar to the values we predict for the epsilon phase. Although simplified significantly, it has been shown that the vacancy formation enthalpy is approximately proportional to the melting point in metallic systems. As such the expected melting point of the epsilon phase will be similar to that of Cr and V, approximately 1900^°^*C*. The compositions with the higher vacancy formation energies (15–25 at.% Mo) are expected, by this measure, to have higher melting points. Future work could also consider the effect of the elements that coordinate with the vacancy that is being formed (in first and second nearest neighbour positions) as some preferred coordinations may lower the vacancy formation energy. The large number of calculations that would be required to carry out this investigation in the HCP system is suited to an empirical bonding investigation of the system rather than using the current method.

The stability of the epsilon phase can be investigated further by understanding the drive for each composition to change stoichiometry, by reducing the amount of Mo content. The following reaction can be considered:

4.5 $${\text{Mo}}_{x}{\text{Ru}}_{7}{\text{Tc}}_{7}{\text{Pd}}_{3}{\text{Rh}}_{3}\to \text{xMo}+{\text{Ru}}_{7}{\text{Tc}}_{7}{\text{Pd}}_{3}{\text{Rh}}_{3},$$

which can be normalized per Mo to give the following reaction:

4.6 $$\text{Mo}{\left({\text{Ru}}_{7}{\text{Tc}}_{7}{\text{Pd}}_{3}{\text{Rh}}_{3}\right)}_{1/x}\to \text{Mo}+{\left({\text{Ru}}_{7}{\text{Tc}}_{7}{\text{Pd}}_{3}{\text{Rh}}_{3}\right)}_{1/x}.$$

**Figure 8.**
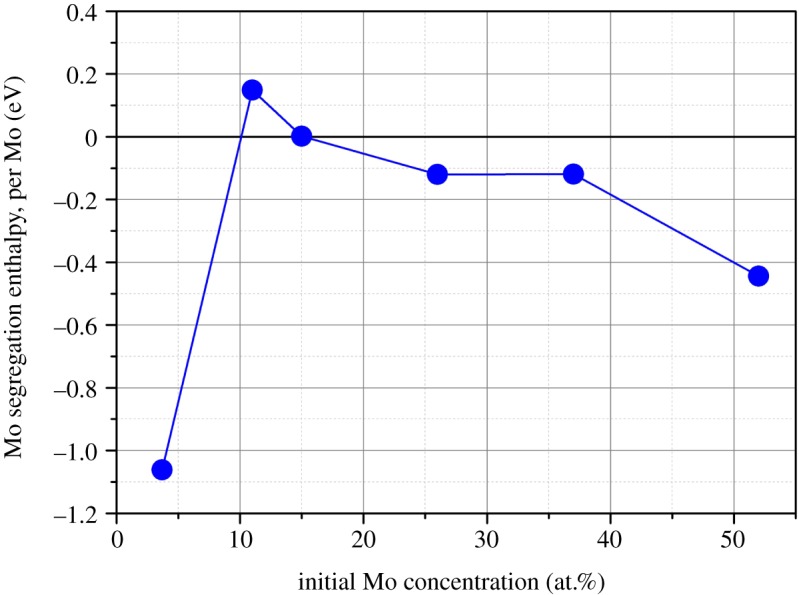
Variation in Mo segregation energy as a function of Mo concentration in Ru_7_Rh_1.5_Pd_1.5_Mo_*x*_ and (Tc_1/3_Ru_2/3_)_7_Rh_1.5_Pd_1.5_Mo_*x*_.

The drive for this segregation reaction to proceed was found to be negligible (close to 0 eV) or unfavourable for all stoichiometries apart from those that contained the highest and lowest concentration of Mo (51.9 at.% and 3.7 at.%, see figure 8). The lower and more favourable segregation energies at the extremes of the compositions considered in figure 8 imply that they are not stable. Configurational entropy can be considered to understand how temperature may affect the drive for Mo segregation. The compositions at the extremes of the Mo content considered remain unstable to temperatures exceeding 2000 K. The other compositions were found to have positive segregation energies (unstable) above 900 K, indicating that the epsilon phase containing approximately 10–40 at.% of Mo is stable. The instability of the phases that contain 3.7 at.% and 51.9 at.% Mo is in agreement with the experimental observations of the system, which predict the formation of the *σ*-phase (high Mo concentrations) or FCC phase (low Mo concentrations). Further work that considers the secondary phases should be carried out to refine the prediction of the maximum and minimum Mo concentration in the epsilon phase.

As oxide fuel is burnt-up, oxygen is released and the formation of UO_2+*x*_ can occur. Highly ionic fission products such as Sr and Ba can react with the excess oxygen initially, but the Mo in the epsilon phase will also react to form BaMoO_3_ or another oxide phase. A simple reaction that can be considered to understand the removal of Mo as a result of the increase in stoichiometry of UO_2_ is provided in the following reaction forming MoO_2_:

4.7 $${\bar{2O}}_{i}+\text{Mo}{\left({\text{Ru}}_{7}{\text{Tc}}_{7}{\text{Pd}}_{3}{\text{Rh}}_{3}\right)}_{1/x}\to {\text{MoO}}_{2}+{\left({\text{Ru}}_{7}{\text{Tc}}_{7}{\text{Pd}}_{3}{\text{Rh}}_{3}\right)}_{1/x}.$$

This reaction is similar to the reaction given in equation (4.6) except that oxygen is available from the uranium dioxide lattice [45,46] (the UO_2_ lattice is denoted by the overbar [5]). The reaction is very exothermic, greater than 7.2 eV in favour for all compositions (when normalized per Mo atom). As a result, any available oxygen will reduce the concentration of Mo in the epsilon phase.

The reduction of the UO_2+*x*_ lattice has major implications for the behaviour of the fuel in the reactor. Accommodation of fission products varies significantly with stoichiometry [10,47], as do the defect transport properties [48] and thermal conductivity of the fuel [49] (among a variety of other material properties).

### 4.3 Accommodation of fission products in the epsilon phase

Although the epsilon phase has been observed to predominantly consist of the five elements considered in this work, a number of other fission products may be associated with the phase. We now investigate the drive for some of these elements to enter the epsilon phase. The fission products considered are the noble gases Kr and Xe; the volatile fission products Cs and I; and the metallic fission products Te, Sn, Cd and Ag—all produced in substantial concentrations as a result of fission processes in ^233^U, ^235^U or ^239^Pu containing fuels [50,51].

Solution and accommodation was initially considered from each element's mono-atomic state (Xe and Kr from their mono-atomic gases, the metals from their isolated metallic form and I in the form of I_2_). Accommodation energies represent the energy required for a species to fill a pre-existing vacancy in the epsilon phase, while solution enthalpies consider the formation of the trap site as well as the extrinsic defect's incorporation. Positive solution and accommodation enthalpies from these states will indicate that a species will not be stable in the epsilon phase, instead forming an alternate phase (in a similar manner to Xe in UO_2_ [7]). A species with a positive solution enthalpy and negative accommodation enthalpy can be considered to occupy a pre-existing defect only and the concentration in the lattice is therefore strictly a function of intrinsic defect formation (in fuels these concentrations will be elevated by radiation processes). Negative solution and accommodation energies for a species indicate that it will readily react into the matrix considered. It should be noted that more complex forms of the fission products are less likely to enter the epsilon phase as these compounds are often more stable (for example, CsI which has a formation energy of −3.56 eV from Cs metal and $\frac{1}{2}{\text{I}}_{2}$). As such, those that have negative solution energies cannot be considered certain to fully react into the epsilon phase.

**Figure 9.**
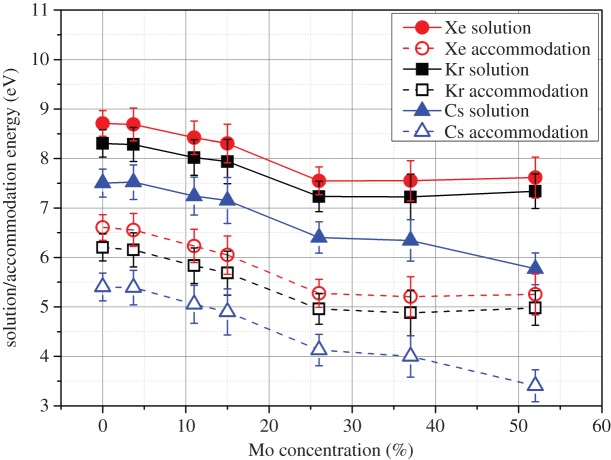
Solution and accommodation energies of Xe, Kr and Cs into the hexagonal epsilon phase with varying Mo concentration. All energies are positive and high indicating extremely limited concentrations.

The solution and accommodation energies for Cs, Xe and Kr were all large and positive, indicating that the epsilon phase will not contain these fission products. Figure 9 reports the solution and accommodation energies of these elements into the epsilon phase as a function of Mo content. All fission products are more soluble when the concentration of Mo in the epsilon phase is higher, although these energies are all very unfavourable.

In the case of Xe and Kr, the positive accommodation and solution energies suggest that these fission products, when injected into the epsilon phase via radiation processes, will either migrate out of the phase or form bubbles within the metal. The concentration of Xe/Kr in the area surrounding the epsilon phase particle in the fuel may be elevated as a result of their insolubility in the phase (assuming a low-energy migration route is present). This increase in concentration may result in the experimentally observed increase in bubble formation associated with the presence of an epsilon phase particle [52].

Cs is not known to form any intermetallics with Mo, Tc, Ru, Rh or Pd so will be likely to react with another fission product (such as I) or oxygen (if an oxide fuel or fuel matrix is being used) inside or outside the epsilon phase. Cs has also reported to be present in the Xe/Kr fission gas bubbles [53].

**Figure 10.**
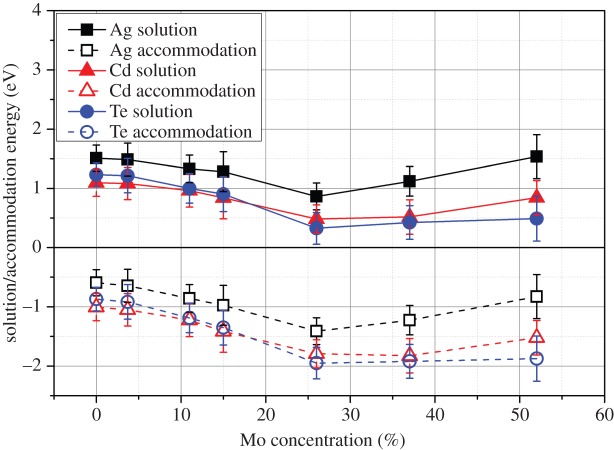
Solution and accommodation energies of Ag, Cd and Te into the hexagonal epsilon phase with varying Mo concentration.

The metallic fission products Te, Cd and Ag all behaved in a similar manner to each other (see figure 10). Each had a moderate to low (positive) solution enthalpy and a negative accommodation enthalpy. Silver solution was least favourable, having a solution enthalpy of 0.86 eV, followed by Cd with a solution enthalpy of 0.48 eV, followed by Te with a solution enthalpy of 0.32 eV. The minimum solution enthalpy for Te, Cd and Ag was at a 25.9% Mo concentration in the epsilon phase. As intermetallics and mixed phases are known to form in the Sn-Te-Pd system and Pd-Ag-Cd system [23] and the volatile Cs_2_Te can also form [54], the solution of Ag, Cd and Te is even less likely to proceed. However, unlike Xe, Kr and Cs, these extrinsic elements can be accommodated in the epsilon phase in small concentrations due to their negative accommodation enthalpy, occupying vacancies caused by radiation events (non-equilibrium vacancy formation) and thermally produced vacancies (equilibrium formation). This prediction is in agreement with the experimental assessment of insoluble residues from McNamara *et al.* [55], who found undetermined concentrations of Te, Ag and I associated with the epsilon phase.

**Figure 11.**
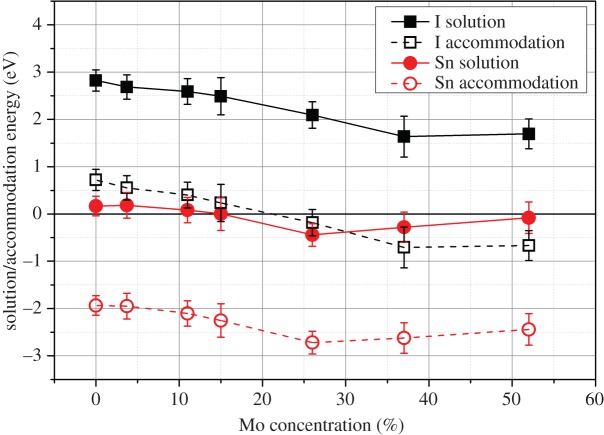
Solution and accommodation energies of I (from I_2_) and Sn into the hexagonal epsilon phase with varying Mo concentration.

Iodine was found to have a positive solution enthalpy from I_2_ gas, and an accommodation enthalpy that was positive for low Mo content epsilon phases but negative for high Mo content epsilon phases (see figure 11). This suggests that I will preferentially fill a pre-existing vacancy in the epsilon phase if introduced to the lattice by a non-equilibrium radiation event in a similar manner to the metals investigated in figure 10. It is known that CsI can form a very stable compound, taking I out of solution from the epsilon phase, but in the absence of Cs or any other reactant the I may remain stable in the epsilon phase until the Mo content is reduced. This release of I may have large implications for fuel performance at higher burnups or during an event that allows for excessive oxidation of fuel such as a clad breach.

Solution energies for Sn are negative for Mo contents in the epsilon phase above 25.9% and positive below, indicating that Sn metal will enter solution into the epsilon phase to some extent unless it reacts with other fission products present or oxygen (see figure 11). All accommodation energies for Sn are very negative (similar to Te, Cd and Ag). As such, Sn accommodation in the epsilon phase is expected in small populations, especially when the oxygen partial pressure is low preventing the formation of the more stable SnO_2_ (the drive for Sn oxidation is similar to that of Mo [23]).

## 5. Conclusion

The epsilon phase has been successfully modelled on the atomic scale using DFT methods. It was shown that including Tc in the alloy has a small but significant effect on the stability of the epsilon phase, increasing the vacancy formation energies and decreasing the expected temperatures at which the epsilon phase is predicted to be stable. Structurally, very little changed by including Tc—only a small increase in both ‘a’ and ‘c’ lattice parameters was predicted.

The Mo content was varied to understand the effect of burnup on the behaviour and stability of the metallic fission product particles. It was shown that at low Mo concentrations of 3.7 at.% and high Mo concentrations of 51.9 at.%, the epsilon phase structure was not stable (preferentially changing stoichiometry to a more stable composition). Even with configurational entropy effects included (up to 2000 K), the extremes of Mo content investigated in this work remained unstable. The other intermediate compositions were shown to be stable with respect to their constituent metals in agreement with past experimental data. Further work should be carried out to compare the hexagonal structure simulated in this work with the FCC phase that is expected to form at low Mo concentrations and the *σ*-phase that is expected to begin to form at high concentrations (more than 50 *at*.%).

The vacancy formation energies were all calculated to be positive, highlighting the stability of the epsilon phase. In both the system that omitted Tc and the system that included the radioactive element, Pd vacancy formation enthalpy was lowest at low Mo concentrations (less than 30 *at*.% Mo), while at greater Mo concentrations Mo itself had the lowest vacancy formation enthalpy. The ease of Pd vacancy formation enthalpy may be a reason for the experimentally observed high vapour pressure of Pd in fast breeder reactor (FBR) fuels (where burnup is high and therefore Mo content in the epsilon phase is low). The Pd in the FBR fuel is often associated with other elements including Pu intermetallics [23]—systems that warrant further investigation in the future.

Oxygen available as a result of the presence of UO_2+*x*_ was found to preferentially react with the Mo in all considered epsilon phase compositions. In this work the formation of MoO_2_ was considered, but other Mo oxides may also form, including MoO_3_ and ternary oxides such as BaMoO_3_.

In a recent paper by Zhang *et al.* [56], it was noted that no HCP high-entropy alloys have so far been described. The metallic phases in this work are consistent with the original description of high-entropy alloys by Yeh *et al.* [57] and the more recent in-depth study by Singh & Subramaniam [58]. We have provided evidence that the epsilon phase (consisting of four or five principal elements) can be considered a high-entropy alloy as the configurational entropy stabilizes the single-phase system.

Calculations understanding the drive for extrinsic defects to be accommodated in the epsilon phase highlighted the varying behaviour that can be expected with a range of fission products. Xe, Kr and Cs were predicted to have very unfavourable solution and accommodation energies and as a result would not enter the epsilon phase structure, instead forming secondary phases either within the epsilon phase particle or outside at a grain boundary. The significant drive against Xe/Kr accommodation and solution may be the cause of the experimentally observed bubble formation in proximity to these metallic precipitates.

Iodine was predicted to have a negative accommodation energy from I_2_ gas into epsilon phases with high Mo contents. This suggests that a measurable amount of I can be held within the epsilon phase until the Mo content is reduced as a result of burnup processes or sudden oxidation of the metallic precipitate. The solution enthalpy for I_2_ into the epsilon phase was positive and as such the concentration will not be large, especially given the likelihood that I will react with other fission products to form more stable compounds.

Tin, tellurium, cadmium and silver were all predicted to behave in a somewhat similar manner. Their accommodation energies were negative for all Mo content epsilon phases, indicating that a small amount of each element could be held within the metallic precipitate. The solution enthalpies were all low, tin having a negative solution enthalpy into high Mo content epsilon phases. As a result, significant concentrations of these elements may be present in the phase. Formation of alternate metallic phases such as the PdAg_*y*_Cs_1+*x*_ (where *x*,*y*≪1 [23]) may drive against solution into the epsilon phase. Future work investigating smaller extrinsic species (such as hydrogen) should also consider the potential drive for accommodation onto interstitial sites in the HCP structure [59].

This work is in good agreement with the experimental effort carried out on this system in the past. The stability, structure and chemical stability all agree well with both work that simulated the metallic phase precipitates (often omitting Tc) as well as those studies that investigated the phase in spent nuclear fuels. It also sets up a great deal more work that should be perused to fully understand the impact of this understudied system that plays a large role in the behaviour of nuclear fuels and waste residues.

## Supplementary Material

5MP_RS.xlsx is a compilation of all of the processed data for the publication.
