# Atomic scale modelling of hexagonal structured metallic fission product alloys

**DOI:** 10.1098/rsos.150193

**Published:** 2015-06-03

**Authors:** S. C. Middleburgh, D. M. King, G. R. Lumpkin

*R. Soc. open sci.* **2**, 140292. (Published online 1 April 2015). (doi:10.1098/rsos.140292)

This correction is to correct an error in equation (4.1). The product should be Mo_6_Pd_7_Rh_7_Ru_34_ not Mo_6_Rh_7_Rh_7_Ru_34_.

The full equation should read as follows:

$$\text{6Mo}+\text{7Pd}+\text{7Rh}+\text{34Ru}\to {\text{Mo}}_{6}{\text{Pd}}_{7}{\text{Rh}}_{7}{\text{Ru}}_{34}.$$

